# iPSC-Derived T and CAR-T Cells of Distinct Phenotypes: Current Protocols, Challenges and Potential for Cancer Treatment

**DOI:** 10.3390/cells15211938

**Published:** 2026-10-25

**Authors:** Margarita Maiak, Anna Efremova, Tatiana Budagova, Diana Mokrousova, Dmitry Goldshtein

**Affiliations:** Research Centre for Medical Genetics, 1 Moskvorechye St., Moscow 115522, Russia; anna.efremova@med-gen.ru (A.E.); workbtu@gmail.com (T.B.); mokrousova@med-gen.ru (D.M.); dvgoldshtein@gmail.com (D.G.)

**Keywords:** induced pluripotent stem cell, T cell phenotypes, adoptive cell therapy

## Abstract

**Highlights:**

1. Autologous CAR-T cell therapy has proven to be an effective method of immunotherapy; yet it has some significant limitations.
2. iPSC technology makes it possible to generate various T-cell phenotypes: from conventional αβ T cells to T cells with innate-like immune properties, such as γδ T, iNKT and MAIT cells.
3. iPSC-derived innate-like T cells represent a promising therapeutic tool for the treatment of solid tumors, as they possess innate properties—including the ability to recognize tumors in an MHC-independent manner, intrinsic cytotoxicity, an enhanced capacity to migrate into tissues, and an ability to reprogram tumor microenvironment
4. Combination therapy with distinct T cell phenotypes may be a promising approach to cancer treatment.

**Abstract:**

Adoptive T-cell therapy has demonstrated substantial clinical efficacy in several hematological malignancies, but autologous manufacturing remains limited by variability in starting cell material, manufacturing time, product heterogeneity, and T-cell dysfunction. Induced pluripotent stem cells (iPSCs) provide a potentially scalable and genetically engineerable source for the generation of standardized allogeneic T-cell products. This review summarizes current approaches to the differentiation of iPSCs into distinct T-cell phenotypes and evaluates their potential for cancer immunotherapy. We first discuss iPSC-derived conventional αβ T cells, including recent advances in feeder-free and scalable differentiation, maturation, and genetic engineering, as well as preclinical and early clinical evidence. We then examine γδ T cells, invariant natural killer T cells, and mucosal-associated invariant T cells, focusing on their distinctive antigen-recognition mechanisms, effector functions, tissue tropism, differentiation strategies, and therapeutic applications. Across these populations, iPSC technology may facilitate standardized and scalable production while enabling targeted engineering of T-cell specificity and functional properties. However, important challenges remain, including efficient lineage-specific differentiation, maturation, phenotypic consistency, long-term persistence, tumor infiltration, and control of alloreactivity and immunogenicity. Overall, iPSC-derived T cells represent a versatile platform for developing diverse cellular immunotherapies, although their clinical potential remains dependent on further technological and translational advances.

## 1. Introduction

According to the World Health Organization, deaths due to malignant neoplasms are one of the leading causes of mortality worldwide [1,2]. Malignant tumors arise, on the one hand, as a result of the acquisition by cells of the ability to proliferate uncontrollably and to evade apoptosis and normal differentiation. These alterations promote the rapid expansion of the tumor cell population, which is characterized by infiltrative and invasive growth. On the other hand, tumor development is associated with a gradual loss of immune control over tumor cells. Tumor evolution is accompanied by the emergence of various mechanisms that enable evasion of immune surveillance. These include the loss of tumor-associated antigens; secretion of immunosuppressive molecules, such as TGF-β, ARG1, IL-10, IDO1, and IL-35; and the establishment of an immunosuppressive tumor microenvironment. The latter is characterized, among other features, by the accumulation of immune cell populations, including myeloid-derived suppressor cells, T_REG_s, Th17 cells, and tumor-associated macrophages, as well as cancer-associated fibroblasts, which impair the effector functions of CTL and NK cells, the major cellular effectors involved in antitumor immunity, and further promote tumor growth [3,4]. In addition, tumor progression is accompanied by systemic inflammation and persistent antigenic stimulation of immune cells, both of which contribute to immune cell exhaustion. Thus, malignant tumors and the immune system engage in a complex network of interactions in which immune cells participate both in antitumor defense and in tumor progression, while tumors not only evade immune surveillance but also promote the accumulation and functional reprogramming of certain immune cell populations through the establishment of a tumor-supportive microenvironment [5].

Cellular immunotherapy is a rapidly developing approach to the treatment of malignant neoplasms that is based on the administration of living immune cells to patients to target and eliminate tumor cells. Adoptive cell therapy (ACT) has emerged as a major area of development, in which leukocytes are isolated, genetically or otherwise modified, and activated ex vivo before being administered to the patient, where they directly participate in antitumor responses. The most widely developed approach is chimeric antigen receptor (CAR) T-cell therapy: as of September 2026, seven CAR T cell products have been approved for the treatment of B-cell malignancies, including lymphomas and leukemias [6,7]. All currently approved products are based on autologous cells, i.e., the patient’s own lymphocytes, which are collected, modified, expanded, and subsequently administered back to the same patient. This therapeutic approach has several limitations and is applicable to a relatively narrow patient population and a limited range of malignancies, primarily hematological cancers. Consequently, new types of cell therapy products are being developed, among which allogeneic products derived from induced pluripotent stem cells (iPSCs) hold considerable promise. Research in this field has traditionally focused on the generation and various modifications of αβ T cells. However, the broad phenotypic diversity of T cells makes it possible to select cell populations with the most favorable characteristics for manufacturing and clinical applications depending on the specific therapeutic objectives. These include CD4+/CD8+ αβ T cells, γδ T cells, invariant natural killer T (iNKT) cells, and mucosal-associated invariant T (MAIT) cells.

## 2. Successes and Limitations of Autologous CAR T Therapy

The clinical efficacy of anti-CD19 and anti-BCMA CAR T cells has been demonstrated repeatedly. However, some patients are unable to receive autologous CAR T cell therapy because of serious contraindications [8,9,10]. For example, this treatment option may be difficult to implement in people living with HIV, as such patients have generally been excluded from large clinical trials of currently approved products. It may also be unavailable to patients with T-cell lymphomas or to those who have previously received CAR T cell therapy [11].

In addition, manufacturing failures may occur even after several attempts and can result from a variety of factors. Common causes of autologous CAR T cell manufacturing failure include low viability of cells collected by leukapheresis, poor cell expansion in vitro, a low final cell dose, and low interferon-γ production by the cells. Manufacturing failure has also been associated with patient characteristics prior to leukapheresis and with the characteristics of the leukapheresis product. For example, a study conducted in a Japanese patient cohort found that manufacturing failure was significantly associated with a lower platelet count in the leukapheresis product, a lower CD4/CD8 T-cell ratio, and higher serum lactate dehydrogenase levels. Important pre-leukapheresis factors included the number of chemotherapy cycles and the time elapsed since autologous stem cell transplantation (in patients who had undergone autologous hematopoietic stem cell transplantation), as well as the number of prior treatment lines and the time elapsed since previous bendamustine treatment. Bendamustine is a chemotherapeutic agent that induces extensive DNA damage and consequently causes cell death. Bendamustine may preferentially affect tumor cells with impaired DNA repair capacity. However, healthy cells, including immune cells, are also affected. Bendamustine treatment has been shown to reduce the number of CD4+ T cells in patients, promote T-cell exhaustion, and impair their proliferative capacity. Collectively, these factors can substantially affect the success of ACT [11,12,13,14].

All currently approved autologous CAR T cell products are based on the use of the predominant T-cell population in peripheral blood: αβ T lymphocytes. Two major types of αβ T lymphocytes can be distinguished based on the presence of the TCR coreceptors CD4 or CD8 on their surface. The contribution of different αβ T-cell subsets to the efficacy of CAR T cell therapy was investigated during the development of autologous CAR T cell therapies. Various approaches have been explored for T-cell selection at the initial stage of CAR T cell manufacturing. One approach involves isolating T cells from the leukapheresis product using the pan-T-cell marker CD3. This results in a mixed T-cell population consisting predominantly of αβ T cells, in which the relative proportions of CD8+ and CD4+ cells may vary between patients, thereby contributing to interpatient variability in the final product. Several studies have demonstrated an association between CAR T cell efficacy and a higher CD4 ratio in the leukapheresis product, or a ratio closer to that observed in healthy individuals (a ratio close to or above 1) [13,15,16]. Conversely, a low CD4 ratio, indicative of a reduced proportion of CD4+ cells, has been associated with poorer treatment outcomes [13,16]. Therefore, a second approach to processing the leukapheresis product for CAR T cell manufacturing is based on the separate isolation of two populations—CD8+ cytotoxic T lymphocytes and CD4+ T helper cells—and their separate expansion and processing in vitro. This approach can substantially reduce product variability compared with products generated from a pan-T-cell-selected population [17].

**Figure 1:**
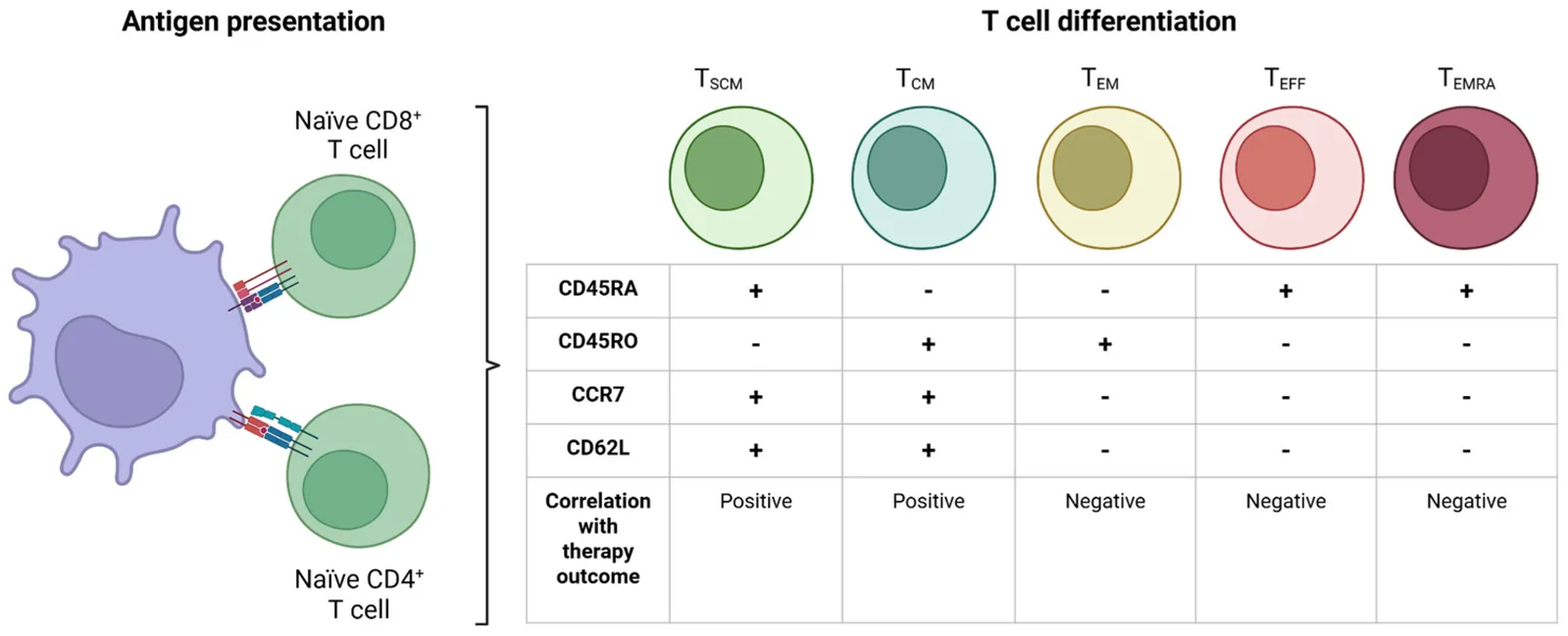
T cell memory and effector subsets. TSCM—stem cell memory T cell, TCM—central memory T cell, TEM—effector memory T cell, TEFF—effector T cell, TEMRA—terminally differentiated effector memory T cells. Created in BioRender. Goldshtein, D. (2026).

Each of the FDA-approved CAR T cell products currently available uses one of these approaches, and both strategies have demonstrated clinical benefit in the treatment of refractory and relapsed B-cell malignancies. Nevertheless, CAR T cell therapy is not effective in all cases, and relapses occur frequently. Relapse may be driven by tumor cell-intrinsic mechanisms, such as antigen escape or antigen loss, but it may also be substantially determined by patient-related factors that vary according to age, sex, previous medical history, the number of prior lines of therapy, and the type of treatment received [18,19]. One possible explanation is an altered composition of the T-cell compartment in patients with malignant neoplasms, characterized by a reduced proportion of naïve T cells and an accumulation of more differentiated T-cell populations, including effector and effector memory cells [20,21]. Treatment failure may also be explained by the persistence of substantial variability in T-cell subsets, which could theoretically persist even in products manufactured from separately isolated CD4+ and CD8+ T-cell fractions [22,23,24]. Both CD4+ and CD8+ T cells can be classified according to their stage of differentiation into naïve, effector, and memory T cells. Memory T cells can be further subdivided into stem cell memory T cells, central memory T cells, and effector memory T cells. Among effector T cells, there is a population of terminally differentiated T cells associated with prolonged antigen exposure (Figure 1) [25,26,27].

All of these cell populations are present in the leukapheresis product and contribute to the final product during CAR T cell manufacturing. In a 2014 study, the contribution of different cellular subsets to the in vivo expansion and persistence of CAR T cells was assessed in 14 patients with lymphoma who received anti-CD19 CAR T cells. The authors found that the population characterized by the CD8+CD45RA+CCR7+ phenotype made the greatest contribution to in vivo persistence. These cells correspond to T_SCM_-like populations [28]. Daniel Sommermeyer et al. evaluated the efficacy of CAR T cell products generated from different CD4+ and CD8+ T-cell subsets. Three CD4+ and three CD8+ T-cell subsets were examined: naïve T cells (T_N_; CD45RO−/CD62L+), central memory T cells (T_CM_; CD45RO+/CD62L+), and effector memory T cells (T_EM_; CD45RO+/CD62L−). Following CAR T cell manufacturing, the authors assessed cytokine production, in vitro cytotoxic activity, and in vivo antitumor activity. CAR T cells derived from naïve T cells demonstrated the highest antitumor activity, followed by those derived from central memory T cells. The authors also evaluated which combinations of CAR T cells generated from different CD4+ and CD8+ T-cell subsets were the most effective. The most effective combinations were CD4+ T_N_ cells with CD8+ T_CM_ cells and CD4+ T_CM_ cells with CD8+ T_CM_ cells [17]. This may be related to the fact that manufacturing CAR T cell products from less differentiated T-cell subsets with greater proliferative potential may result in products enriched in cells with enhanced proliferative capacity and a less differentiated phenotype. Conversely, the use of a mixed T-cell population obtained from heavily pretreated patients, whose T-cell compartment is shifted toward more differentiated subsets, may limit the efficacy of the resulting CAR T cell product or contribute to manufacturing failure [29,30]. In addition, experimental studies have demonstrated that the culture conditions and duration of T-cell cultivation following isolation from patients with lymphoma are important factors determining the phenotype and antitumor activity of cells in the final product. Thus, prolonged ex vivo expansion is associated with a gradual decrease in the proportion of CCR7+ memory cells, an increase in the proportion of cells undergoing effector differentiation, and a decline in the therapeutic properties of the resulting CAR T cells [31]. Collectively, these findings demonstrate experimentally that the initial T-cell phenotype, as well as the conditions and process of ex vivo product manufacturing, substantially influence the efficacy of the final CAR T cell product. Cells with a molecular phenotype characteristic of memory T cells appear to make the greatest contribution to the antitumor activity and in vivo expansion of CAR T cells.

For patients with severe and rapidly progressing disease, the speed with which treatment can be administered is particularly critical. Therefore, the need to repeat the leukapheresis and CAR T cell manufacturing procedures may have a substantial negative impact on the patient’s condition. Thus, the manufacturing of an autologous CAR T cell product can be time-consuming and, in some cases, may not be feasible at all. Another important limitation of autologous CAR T cell therapy is the lack of strict standardization of the final products and administered cell doses, as substantial interpatient variability may be observed in the number of cells administered, their proliferative capacity, persistence following infusion, and the phenotypic characteristics of the resulting CAR T cells. Although product specifications take into account variability in the starting material and the final product between patients, in some cases patients may have to receive products that do not meet the predefined specifications because an in-specification product cannot be manufactured [32].

Another challenge associated with T-cell therapy is the occurrence of adverse events and, consequently, the limited safety of many T-cell-based products for certain patients [33,34]. One of the most common and clinically significant adverse events associated with these therapies is cytokine release syndrome (CRS). This condition can occur following treatment with monoclonal antibodies; for example, it has been reported following administration of the anti-CD3 antibody OKT3, antithymocyte globulin, TGN1412, and rituximab, as well as with T-cell-engaging bispecific antibodies and CAR T-cell therapy [35]. CRS is characterized by massive systemic inflammation resulting from rapid and excessive production of cytokines, including IL-6, IL-10, TNF-α, IFN-γ, and others [36]. In some patients, CRS can be severe and lead to life-threatening complications. CRS is the most common adverse event associated with modified T-cell therapies, particularly CAR T cell therapy; however, patients may also develop ICANS, tumor lysis syndrome, and on-target, off-tumor toxicity [37,38].

**Figure 2:**
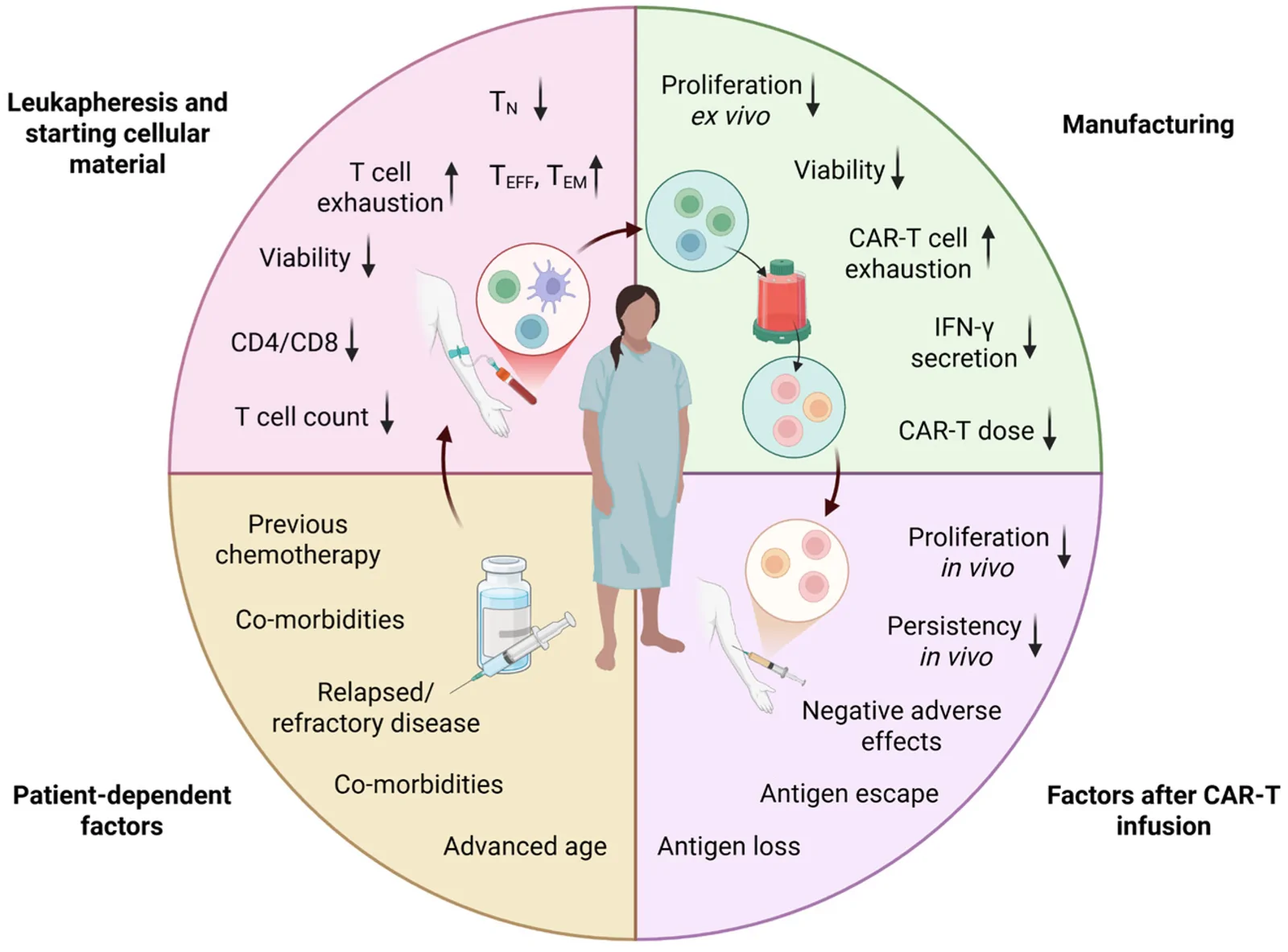
Limitations of autologous CAR T therapy. Arrows a sequence of mutually influencing factors. Created in BioRender. Goldshtein, D. (2026).

Limitations associated with autologous CAR T therapy are presented in Figure 2.

Autologous CAR T cell therapy has established itself as an effective treatment for several malignant diseases. However, challenges remain for a subset of patients, including difficulties in obtaining the therapeutic product, T-cell exhaustion within the cellular product, and complications associated with CAR T-cell therapy. All of these factors can critically influence treatment outcomes in individual patients and highlight the need for further development to improve the safety and accessibility of CAR T-cell products.

## 3. Allogeneic CAR T Therapy

Allogeneic CAR T-cell therapy may provide a means of overcoming the aforementioned limitations [39]. Potential sources of cells for allogeneic T-cell therapy include donor-derived T cells, hematopoietic stem cells (HSCs) obtained from umbilical cord blood, and induced pluripotent stem cells, which can in turn be generated from various types of somatic cells. The number of research studies focused on the development and characterization of allogeneic T-cell therapies, including CAR-T therapies, has been increasing steadily over the past several years. Accordingly, the number of clinical trials evaluating allogeneic T-cell therapy products is also growing. Nevertheless, most of these studies are still in phase I, which primarily focuses on evaluating product safety. Hematological malignancies remain the most extensively studied disease category, although solid malignancies are attracting increasing attention from researchers [40,41,42]. A key feature of developing allogeneic CAR T-cell therapies is the need to introduce additional genetic modifications into the cells beyond the insertion of the chimeric antigen receptor gene. These modifications are required to enhance the safety of the T cells in the recipient by preventing graft-versus-host disease (GvHD) and to enable them to evade immune surveillance by the recipient’s immune system. Additional genetic modifications may also be introduced to enhance tumor targeting, improve infiltration into solid tumors, and increase cell persistence, proliferation, and functional activity [42].

### 3.1. iPSC-Derived Allogeneic T Cell Therapies

A promising direction in the field of allogeneic T-cell therapy is the development of T cells derived from iPSCs. Such approaches may help overcome a number of limitations associated with autologous T-cell therapy. iPSCs can be subjected to extensive genetic engineering and, in principle, may serve as a renewable source of T cells for the treatment of a wide range of infectious and malignant diseases. In addition, in the context of malignant neoplasms, tumor development and progression are accompanied by persistent antigenic stimulation of the immune system, resulting in profound functional impairment of multiple immune cell populations, including components of the adaptive immune system and T cells. In some cases, failure or suboptimal efficacy of autologous CAR T-cell therapy has been associated with the functionally exhausted state of T cells obtained from patients. iPSCs, in contrast, may provide a source of cells with a less differentiated phenotype and without the exhaustion acquired during prolonged antigenic stimulation. A high proportion of such cells in existing T-cell products has been associated with successful antitumor therapy [15,43,44].

The generation of iPSCs was first described in 2006. Shinya Yamanaka and Kazutoshi Takahashi identified four key transcription factors (Oct3/4, Klf4, Sox2 and c-Myc), the introduction of which into somatic cells reprograms them into a pluripotent state. The authors demonstrated that transduction of somatic cells (mouse tail-tip fibroblasts) with retroviral vectors encoding these factors enabled the generation of induced pluripotent stem cells. Following subcutaneous transplantation into nude mice, these cells formed tumors containing tissues derived from all three germ layers, thereby confirming their pluripotent state [45]. In 2007, the authors successfully applied the developed reprogramming method to adult human fibroblasts, generating cells that resembled human embryonic stem cells in terms of morphology, proliferative capacity, surface antigen markers, and gene expression; the epigenetic status of pluripotency-associated genes; and telomerase activity [46]. Subsequent studies by different research groups demonstrated that iPSCs could be generated not only from fibroblasts but also from other types of somatic cells, including PBMCs, and that iPSCs could be directed to differentiate into various tissue and cell types. In addition, a range of new and potentially safer approaches for reprogramming somatic cells into pluripotent stem cells has been developed. Thus, currently available methods for iPSC generation include both viral approaches (integrating and non-integrating) and non-viral approaches, such as episomal plasmids, mRNA, and small molecules. Among the methods currently considered standard and relatively safe are the use of non-integrating Sendai virus vectors and episomal plasmids, both of which were introduced in 2009 [47,48].

In 2009, it was demonstrated that iPSCs could be differentiated toward the T-cell lineage. A US-based research team built on earlier findings showing that human HSC derived from umbilical cord blood, as well as embryonic stem cells, could differentiate toward T lymphocytes when cultured on OP9-DL1 cells—a murine cell line derived from mouse embryonic bone marrow stromal cells and producing the DLL1 (Delta-like ligand 1) ligand [49,50]. The authors used a mouse iPSC line (iPS-MEF-Ng-20D-17) generated from mouse embryonic fibroblasts. The iPSCs were cultured on OP9 cells bearing the DL1 ligand for three weeks. The resulting differentiated population consisted predominantly of various subsets of immature thymocytes, including both double-positive (DP, CD4+CD8+) and single-positive (SP, CD4−CD8+) cells. The authors reported that adoptive transfer of the resulting cells into Rag-deficient mice restored the T-cell compartment: four weeks after intravenous administration, flow cytometric analysis of single-cell suspensions from the lymph nodes and spleens of the recipient mice detected the T-cell surface markers CD4, CD8, CD3, and TCRβ [51]. Thus, this study demonstrated the feasibility of directed differentiation of iPSCs toward the T-cell lineage. Importantly, when iPSCs generated from fibroblasts are differentiated toward the T-cell lineage, the resulting T cells develop from cells whose TCRs have not undergone the critical stages of differentiation and selection that normally occur in the thymus in vivo. In particular, these cells have not undergone positive and negative selection, which shapes the T-cell repertoire according to the affinity of TCRs for self-antigens. The absence or incomplete recapitulation of these thymic selection processes represents an important consideration for the safety of iPSC-derived T cells intended for cellular immunotherapy.

The generation of T cells with defined antigen specificity from iPSCs was first described in 2013. In this study, iPSCs were generated from mature, clonally expanded antigen-specific CD8+ T cells obtained from an HIV-1-infected patient. Nishimura et al. isolated CD8+ T cells specific for the HIV-1 Nef protein epitope Nef-138-8(WT) and subsequently transduced these cells with retroviral vectors encoding OCT3/4, SOX2, KLF4, c-MYC, NANOG, and LIN28A to reprogram them into a pluripotent state. The authors subsequently generated mature CD8+ T cells and demonstrated their functional activity and high proliferative potential. Thus, Nishimura et al. were the first to generate rejuvenated, functionally active cytotoxic T cells that retained a T-cell receptor specific for a defined antigen [52]. One of the key conclusions of the study was that this technology could potentially be used to generate “rejuvenated” T cells. The immune system undergoes profound age-associated changes, and alterations in T-cell populations are among the important factors contributing to immune dysfunction in older individuals. These changes are accompanied by increased expression of genes coding proteins such as TIM-3 and PD-1 on the T cell surface, which are commonly used as markers associated with T-cell exhaustion. In addition, prolonged antigenic stimulation associated with chronic infectious diseases and malignant neoplasms can promote T-cell exhaustion and increased production of inhibitory receptors belonging to immune checkpoint pathways. T-cell exhaustion is one of the factors that may contribute to the failure of ACT and has therefore been the subject of extensive experimental research aimed at developing T-cell products enriched in less differentiated T-cell subsets and memory populations [53]. In their study, Nishimura et al. demonstrated that mature T cells can be reprogrammed into a pluripotent state and subsequently redifferentiated into T cells with a less exhausted, “rejuvenated” phenotype. Following this process, the cells exhibited high proliferative activity while retaining effector functions, including cytotoxicity, interferon-γ production, and degranulation [52]. Thus, this work provided an early demonstration of an important potential advantage of iPSC-derived T cells: the ability to generate T-cell products with a less differentiated and less exhausted phenotype and with a high proportion of cells exhibiting characteristics associated with younger T-cell subsets. These properties may be particularly advantageous for ACT, in which the differentiation state, proliferative capacity, persistence, and functional fitness of the administered T cells can critically influence therapeutic efficacy [54].

Numerous subsequent studies by different research groups have addressed a broad range of questions related to the application of iPSC technology to T cells, including the development of different protocols for T-cell generation and the integration of iPSC technology with approaches for targeting tumor-associated antigens, including the generation of iPSC-derived CAR T cells [55]. Active research is also being conducted to modify iPSC-derived T cells to target other types of hematological malignancies as well as solid tumors. However, these approaches have not yet resulted in broadly established clinical applications, and no autologous CAR T-cell products targeting other types of hematological malignancies or solid tumors have been approved by the FDA to date. Many studies have also focused on generating distinct T-cell subsets rather than being limited to CD4+ or CD8+ αβ T cells [55]. In this context, the potential of other T-lymphocyte subsets for ACT of malignant neoplasms is becoming increasingly relevant, particularly because some of these populations may offer biologically distinct properties that could make them suitable for specific therapeutic applications. Since αβ T cells constitute the predominant T-cell population in peripheral blood, obtaining other T-cell subsets in quantities sufficient for autologous cell therapy can be a highly labor-intensive process. iPSC technology may therefore help address several challenges simultaneously: generating a specific T-cell subset required for therapy; obtaining that subset in sufficient quantities for therapeutic administration; standardizing the resulting T-cell product; and controlling key safety-related characteristics of the product [56].

### 3.2. αβ T Cells in Cancer Immunotherapy

In CAR T-cell therapy, αβ T cells are the most extensively validated T-lymphocyte population. The CAR T-cell products approved to date are predominantly based on this cell type, with the most established clinical results obtained in B-cell malignancies bearing the tumor-associated antigens CD19 and BCMA.

As discussed above, the manufacture of autologous CAR T-cell products is associated with numerous challenges. Consequently, various approaches to allogeneic αβ T-cell therapy are currently being developed, including approaches based on iPSC technology. The use of iPSC technology followed by directed differentiation toward the αβ T-cell lineage, combined with additional genetic modifications, may enable the generation of a more universal cell and off-the-shelf therapy product with a potentially improved safety profile, enhanced persistence, and a less differentiated phenotype.

#### 3.2.1. Differentiation of iPSCs into αβ T Cells: Limitations and Challenges

Several approaches to the differentiation of iPSCs into T cells have been developed to date. A classical approach involves co-culturing iPSCs with murine stromal cells bearing the Notch ligands DLL1 or DLL4 on their surface, as described in detail above [50,57,58]. To eliminate the need for feeder cells, culture surfaces coated with Notch ligands have also been developed. Various soluble factors that mimic signals provided by the thymic niche are also used during differentiation, including IL-7, FLT3L, SCF, and, in some protocols, CXCL12, CCL25, and others. IL-7 promotes cell survival, proliferation, and differentiation, FLT3L promotes proliferation, and SCF supports the proliferation of double-negative (DN) lymphoid progenitors [59]. A major limitation of feeder-based approaches is their limited suitability for clinical translation because they rely on cells of murine origin. Different feeder cell types are required at different stages of differentiation: MEFs for iPSC expansion, OP9 or C3H10T1/2 cells for induction of differentiation toward hematopoietic progenitors, and OP9-DLL1 or OP9-DLL4 cells for T-cell differentiation. Different culture media are also required at different stages [60]. Another limitation of conventional 2D differentiation approaches is that they frequently result in the generation of incompletely mature CD5-/low T cells and CD5+CD7+ progenitor populations. Consequently, additional maturation steps are often required to promote the development of CD8 and CD4 SP T cells. CD8 SP cells can be generated by stimulating DP cells with anti-CD3 antibodies, whereas the generation of CD4SP cells requires a more complex approach, as discussed below [61].

An additional challenge associated with some of the 2D differentiation protocols is the propensity of the resulting cells to acquire an innate-like phenotype, including the emergence of populations phenotypically resembling NK cells or γδ T cells. In particular, instead of mature CD8αβ+ T cells, cultures may become enriched in CD8αα+ cells as well as T cells producing NK-cell-associated markers, including CD56 [54,62]. This feature necessitates further optimization of differentiation protocols to shift the development of iPSC-derived T cells away from an innate-like state toward conventional T cells with a CD8αβ+CD5+CD56− phenotype. In some studies, the emergence of this phenotype has been linked to the characteristics of TCR and CAR signaling, particularly when iPSCs are engineered to constitutively express a CAR-coding gene. One potential strategy for addressing this issue is to eliminate endogenous *TCR* expression in iPSCs and introduce the CAR gene when the cells have reached the DP stage [63,64]. Another proposed approach is to modify cytokine signaling by using a defined combination of cytokines, including IL-7, IL-15, IL-12, IL-18, IL-21, and TL1A, to promote differentiation toward conventional T-cell phenotypes [54]. Mature SP T cells have also been generated using artificial thymic organoids. However, this approach requires the use of feeder cells, which limits its clinical translation and scalability [64,65].

In a 2021 study, Shoichi Iriguchi and colleagues aimed to address two major limitations of previously developed protocols for differentiating iPSCs into CD8αβ T cells: low differentiation efficiency and poor scalability. To induce hematopoietic differentiation of T-iPSCs, the authors developed a serum-free and feeder-free system. Cells were seeded onto ultra-low-attachment plates and incubated under hypoxic conditions for 24 h. To induce differentiation into mesoderm and subsequently into hematopoietic progenitors, the cells were cultured in the presence of CHIR99021, BMP-4, bFGF, and VEGF, with SB431542 added on day 2 of culture. By day 14, this approach yielded a substantial population of CD34+/CD43+ cells. T-cell differentiation was performed on plates coated with recombinant human DL-4 (rhDL-4), thereby eliminating the need for feeder cells, and the differentiation medium was supplemented with SCF, TPO, FLT3L, and IL-7. The cells were cultured under these conditions for 21 days, and the authors demonstrated that the system reproduced the major stages of thymocyte development, from CD7+ cells through CD5+ cells to CD4/CD8 DP cells. The authors also tested additional factors involved in T-cell differentiation and found that the combination of CXCL12, also known as stromal cell-derived factor 1 (SDF-1), and SB203580, a p38 inhibitor, produced a synergistic positive effect. This combination simultaneously increased the yield of DP cells and immature CD8+ cells while reducing the frequency of undesirable CD4−CD8− DN cells and prematurely generated CD8 SP cells.

To induce the maturation of T-cell progenitors into mature CD8+ T cells, the researchers stimulated the progenitor cells with anti-CD3 antibodies in the presence of IL-7 and IL-2. The resulting iCD8αβ lymphocytes exhibited a high proliferative capacity. When the cells were stimulated with a combination of several soluble factors, including IL-7, IL-15, IL-21, IL-12, and IL-18 as well as TL1A, the researchers achieved approximately 400-fold expansion within 14 days using a feeder-free and serum-free culture system. Overall, the researchers were able to achieve a total expansion during T-cell differentiation of 5166-fold over 35 days. At the end of the differentiation culture, by day 42, they expected to obtain approximately 1.25 × 10^9^ mature iCD8αβ T-cells. Additionally, the platform proved to be compatible with CAR engineering [60]. Nevertheless, an important concern is the persistence of the risk of GvHD, as the resulting cells retain an endogenous TCR. Therefore, the development of a fully applicable iαβ T-cell product requires additional genetic modifications beyond CAR gene insertion, including HLA editing, TCR disruption or truncation, and the introduction of additional genes that enable the cells to evade immune surveillance by the recipient’s immune system. Moreover, the authors did not develop a similarly comprehensive and scalable approach for generating iCD4αβ T lymphocytes.

Other feeder-free systems for generating iT cells have also been developed, with potential for clinical translation and compatibility with CAR engineering. One such approach involves the use of culture surfaces coated simultaneously with DLL4, which promotes T-cell specification, and VCAM1, a cell adhesion molecule that enhances this process. This combination has been proposed for use both during the endothelial-to-hematopoietic transition, where it enhances commitment toward the T-cell lineage, and during subsequent T-cell differentiation. The combination of these proteins was shown to synergistically activate Notch signaling and enabled the generation of CD8αβ+CD4−CD3+TCRαβ+ T cells under feeder-free and serum-free conditions [66]. In addition to protein-coated culture surfaces, DLL4-coated microbeads have been proposed as an alternative approach. This strategy can overcome the surface-area limitation associated with using protein-coated plates and may therefore facilitate more efficient scaling of the differentiation process [67].

The maturation of iDP cells into iCD4SP T cells remains a more challenging task. Early attempts to generate CD4SP T cells from DP cells involved the artificial introduction of a gene encoding CD4, which substantially complicated the generation and scalability of this cell population [68]. Experiments using an artificial thymic organoid (ATO)-based feeder system for iPSC differentiation demonstrated that CD4+ cells could be generated alongside CD8+ T cells, although their yield turns out to be substantially lower [69]. In 2024, a feeder-free approach for generating iCD4+ T_REG_s was proposed using stimulation with PMA and ionomycin [70]. In a 2026 study, Julian Amirault et al. demonstrated that Notch signaling becomes dispensable for the maturation of CD4SP T cells from committed DP progenitors in an iPSC-based system. Using a feeder-free system that did not require the formation of ATOs, the authors showed that attenuation of Notch signaling promoted differentiation toward a CD4+ phenotype [71]. Other approaches have also been proposed that combine the modulation of Notch, TCR, and integrin signaling to control CD4SP T-cell differentiation [72].

#### 3.2.2. Preclinical and Clinical Studies of iαβ T Cells

The efficacy of iT and iCAR T cells against both solid and hematological malignancies has been repeatedly demonstrated in preclinical in vivo models. In a study by Hidehito Saito and colleagues, the efficacy of antigen-specific iCD8+ T cells was demonstrated in a syngeneic melanoma model. Following adoptive transfer into mice and additional intraperitoneal administration of IL-2, the cells induced regression of established melanoma tumors. Surviving animals subsequently developed antigen-specific immunological memory [73]. Similarly, the first human CD19-CAR T cells generated from T-iPSCs effectively suppressed the growth of CD19+ lymphoma in a xenograft model [62]. Subsequent studies demonstrated that iPSC-derived CD20 CAR T cells with *B2M* (gene encoding β2-microglobulin), *CIITA*, and *PVR* knockout and engineered expression of *HLA-E* retained antitumor activity while exhibiting increased resistance to allogeneic immune responses [74]. The proliferation and persistence of iCD8αβ cytotoxic CAR T cells within tumors can also be enhanced by knockout of the genes encoding DGKα and DGKζ combined with the introduction of a gene encoding membrane-bound IL-15/IL-15Rα. In vivo, these cells demonstrated antitumor efficacy comparable to that of primary CD8+ CAR T cells [75].

Immunotherapeutic T-cell products generated from iPSCs remain predominantly at the preclinical stage of development. The first clinical administration of CD19 iCAR T cells was performed in patients with relapsed/refractory B-cell lymphomas, chronic lymphocytic leukemia, and B-cell acute lymphoblastic leukemia [76]. At least four clinical studies of iT-cell products in patients with hematological and solid malignancies are currently at early stages of clinical development and are summarized in Table 1. Importantly, for clinical application and the development of universal products, iPSC-derived T cells, including CAR T cells, require additional modifications aimed at reducing alloreactivity and immunogenicity. One of the most commonly used strategies to reduce the risk of GvHD is the disruption of endogenous TCR, while one of the most common strategies for prevention of host-mediated rejection is disruption of the gene encoding B2M, which results in loss of HLA class I because B2M is required for the stable production of MHC class I molecules. However, loss of HLA class I can trigger a “missing-self” response mediated by NK cells. To overcome this barrier, several strategies have been proposed for engineering iT cells, including overexpression of *HLA-E*, the product of which interacts with CD94/NKG2A on NK cells; *HLA-G1*, which interacts with the KIR2DL4 receptor on NK cells; and *CD47*, which binds SIRPα on macrophages and inhibits phagocytosis, thereby providing a “don’t-eat-me” signal. Disruption of the *CIITA* gene leads to loss of MHC class II gene expression, as CIITA is the master regulator of MHC class II gene expression [77,78].

Thus, preclinical studies have already demonstrated that iPSC-derived αβ T cells can exert potent antitumor effects in vivo, including in models of both hematological and solid malignancies, while early clinical studies have provided initial evidence supporting the feasibility of using these cells in patients. At the same time, several major challenges remain unresolved, including achieving long-term persistence, ensuring effective infiltration and function within solid tumors, and preventing immune rejection of allogeneic, “off-the-shelf” products. The latter challenge requires additional genetic modifications aimed at reducing alloreactivity and immunogenicity.

### 3.3. γδ T Cells in Cancer Immunotherapy

γδ T lymphocytes are a unique and heterogeneous population of T cells whose T-cell receptor (TCR) consists of γ and δ chains. These cells are among the first T-cell populations to develop in the embryonic thymus and represent a unique population at the interface between innate and adaptive immunity. The mechanisms of thymic selection of γδ T cells differ substantially from the classical MHC-dependent positive selection of αβ T cells, and γδ T cells rapidly migrate from the thymus to peripheral tissues, including the epidermis, mucosal surfaces of various organs, and secondary lymphoid organs. Consequently, during embryonic development, these cells gradually cease to be the predominant T-cell population in the thymus. In adults, they represent a relatively minor population both in the thymus, where they account for approximately 1% of developing T cells, and in peripheral blood, where they constitute approximately 5% of lymphocytes [81,82]. Most peripheral γδ T lymphocytes lack the CD4 and CD8 coreceptors, and consequently, a substantial proportion of these cells recognize antigens in an MHC-unrestricted manner [83,84]. A subset of γδ T cells has the CD8αα homodimer. γδ T cells can recognize MHC class I chain-related proteins (MIC) molecules and pathogen-associated molecular patterns through specialized receptors present on the surface of some γδ T-cell subsets, including NKG2D, NKG2/CD94, and Toll-like receptors (TLRs) [84,85,86,87]. Thus, these cells exhibit both T-cell and NK-cell-associated phenotypic and functional features. In addition, under certain conditions, γδ T cells can also perform antigen-presenting functions [88].

The extensive phenotypic diversity of γδ T lymphocytes is accompanied by substantial functional heterogeneity, including the presence of both pro-tumor and antitumor γδ T-cell populations. Their tissue residency in numerous anatomical sites throughout the body, including the epidermis and dermis of the skin, uterus, testes, intestine, adipose tissue, meninges, liver, and spleen, indicates an intrinsic tropism for different tissues and makes γδ T cells promising effector cells for the treatment of solid tumors [82]. Their potential as therapeutic effectors is further supported by their rapid activation, a relatively low risk of GvHD in the allogeneic setting due to MHC-unrestricted antigen recognition, and the combination of effector functions associated with both adaptive immunity (TCR-mediated activation) and innate immunity. The latter include activation through NK-cell-associated receptors such as DNAM-1, NKG2C, NKp44, NKp30, and NKG2D, as well as the ability to recognize the stress-induced molecules MICA and MICB. γδ T cells can also recognize non-peptide antigens, and some γδ T-cell subsets can produce functional CD16, enabling the use of antibody-dependent cellular cytotoxicity (ADCC) as an additional effector mechanism in therapy [89]. During human ontogeny, three major γδ T-cell populations can be distinguished based on their Vδ chains: Vδ1, Vδ2, and Vδ3. The most common Vγ chains include Vγ2, Vγ3, Vγ4, Vγ5, Vγ8, Vγ9, and Vγ11 [90]. In the peripheral blood of most individuals, Vδ2+ cells constitute the predominant γδ T-cell population and are also the first major γδ T-cell population to emerge during ontogeny [91]. The Vδ2 chain most commonly pairs with Vγ9, making Vγ9Vδ2 cells the predominant Vδ2+ population. A unique feature of these cells is their ability to recognize phosphoantigens, such as HMBPP, which is present in many pathogens, and IPP, whose accumulation is characteristic of certain tumor cells [92]. Vδ1+ T cells account for approximately one-third of γδ T cells in peripheral blood, while the majority of these cells reside in peripheral tissues [91,93]. These cells exhibit substantial clonal diversity and can recognize lipid antigens presented by the MHC class I-like molecules CD1d and CD1c. Vδ3+ cells are the rarest and least abundant of the three major populations and are found predominantly in the liver and intestine. Individual clones of these cells have been shown to recognize annexin A2, MR1, and lipid antigens presented by CD1d [94]. All three populations can be further subdivided into numerous phenotypic and functional subsets that perform diverse functions in peripheral tissues and participate in tumor-related processes in different ways.

One of the first observations linking γδ T cells to antitumor immunity was the discovery that aminobisphosphonates can activate and selectively expand Vγ9Vδ2+ T cells. Aminobisphosphonates, including pamidronate and zoledronic acid, are used in the treatment of malignant diseases to inhibit osteoclast-mediated bone resorption, including in patients with bone metastases. It was subsequently demonstrated that, in patients with malignancies, these drugs can induce the activation and proliferation of Vγ9Vδ2 T cells. This effect is associated with the influence of aminobisphosphonates on the mevalonate pathway: inhibition of this pathway leads to the accumulation of phosphoantigens in target cells, including tumor cells, which in turn activates Vγ9Vδ2 T cells through the BTN3A1/BTN2A1 complex. Thus, pharmacological inhibition of the mevalonate pathway can induce a signal resembling that generated when Vγ9Vδ2 T cells recognize metabolically activated or transformed cells [95,96].

It is now well established that γδ T cells can play diverse roles in the tumor microenvironment. Some γδ T-cell populations can exert immunoregulatory functions through cytokine secretion, whereas others can mediate cytotoxicity; depending on their phenotype and functional state, γδ T cells may therefore have either pro-tumorigenic or antitumor effects [97]. Several studies have shown that a higher abundance of γδ T lymphocytes in certain tumors, including colorectal cancer, bladder cancer, and hepatocellular carcinoma, may be associated with a favorable prognosis [98,99,100]. At the same time, other studies have demonstrated the opposite association. In solid tumors, Vδ1+ cells are the most frequently represented γδ T-cell subset, and studies have demonstrated substantial functional plasticity of these cells with respect to their pro-tumorigenic and antitumor properties. For example, a recent study found that a high abundance of peripheral Vδ1+ cells in patients with melanoma was associated with poorer survival, whereas a higher abundance of Vδ1+ T cells within the tumor was associated with more favorable survival outcomes [101]. In malignant tumors, Vδ1+ cells can acquire pro-tumorigenic phenotypes with functions resembling those of CD4+ T_REG_s and Th17 cells. These cells produce cytokines associated with tumor progression, including IL-4, IL-17, IL-10, and TGF-β. In a 2014 study, γδ T cells isolated from the blood of healthy donors and patients with neuroblastoma were successfully expanded in vitro using antigen-presenting cells bearing antibodies against the corresponding γδ TCRs. Three populations of expanded cells were identified: Vδ2+, Vδ1+, and cells expressing other Vδ-chain TCR subtypes (Vδ1-Vδ2-). All three populations exhibited cytotoxicity against tumor cells. Vδ2+ cells were particularly effective in mediating CD16-dependent antibody-dependent cellular cytotoxicity (ADCC), whereas Vδ1+ cells demonstrated predominantly antibody-independent cytotoxicity [102]. γδ T cells with antitumor phenotypes more commonly belong to the Vδ2+ subset and produce perforin and granzymes, as well as pro-inflammatory cytokines such as IFN-γ and TNF-α. In addition, a proportion of antitumor γδ T cells can mediate ADCC and present tumor antigens to CTLs and T helper cells, thereby promoting their activation and recruitment to the tumor site and contributing to the synergistic enhancement of antitumor immunity [88,102,103].

Overall, the tropism of γδ T lymphocytes for barrier and peripheral tissues, together with the demonstrated antitumor activity of certain γδ T-cell subsets, makes these cells a promising platform for CAR-based immunotherapy. The feasibility of isolating Vγ9Vδ2+ cells from peripheral blood and expanding them ex vivo has already been demonstrated in vitro. Their antitumor activity has also been demonstrated in the laboratory as well as in clinical settings [89].

At the same time, γδ T lymphocytes constitute no more than approximately 5% of peripheral blood lymphocytes in healthy individuals, and their abundance in peripheral blood may be reduced in certain malignant diseases. In addition, these cells may acquire an exhausted phenotype in the context of malignancy [91]. The ex vivo expansion of primary γδ T cells from peripheral blood can therefore be challenging because of T-cell exhaustion and substantial donor-to-donor variability in fold expansion. Moreover, the number of cells obtained using this approach may be sufficient only for autologous applications. The cell yields achievable by this method may be insufficient for the large-scale manufacturing of allogeneic γδ T-cell products. Therefore, the generation of iPSC-derived γδ T (iγδT) cells represents a promising research direction.

#### 3.3.1. Differentiation of iPSCs into γδ T Cells: Limitations and Challenges

Primary γδ T cells, as well as somatic cells that are not of the T-cell lineage, can be used as starting material for generating iPSCs for subsequent differentiation into iγδT cells. The main advantage of using γδ T cells as the starting material is that their genome already contains rearranged γ- and δ-chain TCR loci generated through V(D)J recombination. Following reprogramming, γδ T cells acquire a pluripotent state, while the pre-existing TCR gene rearrangements are retained. Therefore, γδ T-cell-derived iPSCs can serve as a source of T cells with predefined TCR specificity, without the need to recapitulate TCR rearrangement de novo during differentiation. In contrast, when iPSCs derived from other types of somatic cells are used, the generation of γδ T cells requires obtaining the appropriate rearrangement and expression of the γδ TCR genes during differentiation or introducing TCR genes with a defined specificity. Both approaches can further complicate the differentiation process. The isolation of primary γδ T cells from PBMCs for subsequent iPSC generation can be facilitated by in vitro enrichment with zoledronate and IL-2. This combination of factors promotes the activation and proliferation of γδ T cells isolated from peripheral blood, thereby facilitating the subsequent selection of γδ T-cell clones for reprogramming [104].

Ontogenetically, γδ T cells develop earlier than αβ T cells. Whereas αβ T cells develop exclusively from double-positive (DP) thymocytes, γδ T cells can develop at both the DN and DP stages, with progression through the DP stage not being essential for their development. In both cases, early specification toward the T-cell lineage depends on Notch signaling. The early stages of γδ T-cell generation largely overlap with approaches used for αβ T-cell differentiation. Following the induction of mesodermal differentiation and formation of hemogenic endothelium, CD34+/CD43+ hematopoietic progenitors are isolated and subsequently directed toward the T-cell lineage in the presence of the Notch ligand DLL1 and cytokines such as Flt3L, SCF, and IL-7. These conditions primarily promote the transition of iPSCs toward the T-cell lineage but do not, by themselves, determine the final αβ or γδ lineage specification. Further differentiation toward the γδ T-cell lineage does not require progression through the DP stage. The initiation of γδ TCR gene expression and subsequent commitment toward the γδ lineage are supported by signaling through the γδ TCR. Accordingly, once T-cell progenitors have been generated, protocols for γδ T-cell differentiation are designed to promote and maintain γδ T-cell specification and maturation through the use of specialized factors.

In a 2023 study, Murai and colleagues performed the early stages of differentiation using an OP9-DLL1 feeder system supplemented with a combination of soluble factors, including Flt3L, SCF, IL-7, TPO, and ascorbic acid. Following T-lineage specification, the cells were transferred to a feeder-free system containing 2-mercaptoethanol, IL-2, and HMBPP, a phosphoantigen capable of stimulating the Vγ9Vδ2 TCR. The resulting γδ T cells exhibited cytotoxicity against several tumor cell lines and phenotypically resembled a minor Vγ9Vδ2+ population found in peripheral blood. Based on their CD2-negative and CD7-positive phenotype, the cells also resembled γδ T cells found in fetal thymus. In addition, the iγδT cells were CD5-negative, a phenotype that has been associated in other studies with greater cytotoxicity than that observed in CD5-positive γδ T cells [105,106].

γδ T cells are also considered promising candidates for immunotherapy because they express genes for activating receptors of the NKR family, including NKG2D, and recognize antigens in an HLA-independent manner. Therefore, in principle, CAR engineering of γδ T cells may provide broader mechanisms of tumor recognition than those available to conventional αβ T cells. Even when equipped with a CAR, αβ T cells may become ineffective following antigen escape or antigen loss, which are among the mechanisms that can contribute to relapse after CAR T-cell therapy [89,107,108]. In 2019, a strategy for generating so-called “mimetic” γδ T cells was proposed. Unlike protocols designed to reproduce the classical developmental pathway of γδ T cells, this study used a differentiation protocol originally developed for generating iNK cells. Hematopoietic differentiation of iPSCs was performed on OP9 cells for 12 days. The differentiated cells were then transferred onto OP9-DLL1 cells, which express a Notch ligand gene, to induce lymphoid differentiation in the presence of SCF, FLT3L, IL-7, and IL-15. By day 33 of culture, suspended lymphoid cells emerged, 53% of which were γδTCR+CD3+. These cells were Vγ9+Vδ2+ and also produced the NK-cell marker CD56; based on surface marker analysis, 45% of the cells were CD3−CD56+. The authors referred to these cells as “mimetic” γδ T cells. Compared with conventional γδ T cells, they produced a broader repertoire of activating NK-cell receptors, as well as CD16 and the apoptosis-inducing ligands TRAIL and FasL [109]. This protocol therefore enables the generation of iγδT cells with several features that may be advantageous for the development of universal cell therapy products. First, introduction of a CAR could provide these cells with defined antigen specificity. Second, their CD16 production could allow them to be used in combination with therapeutic antibodies through CD16-mediated ADCC. Third, they retain an endogenous Vγ9Vδ2 TCR capable of recognizing phosphoantigens in an HLA-independent manner. Fourth, the cells were reported not to induce GvHD. Finally, their expression of NK-cell receptors provides additional mechanisms for tumor recognition and activation that are characteristic of innate immunity.

A limitation of the systems described above is the need for feeder cells and serum-containing culture media. To overcome these challenges, feeder-free and serum-free differentiation protocols have been developed. T-lineage differentiation can be performed on culture plates coated with VCAM1 and DLL4 using serum-free media and supplements in the presence of DKK-1 and azelaic acid. In this system, DLL4 mediates Notch activation, whereas VCAM1 promotes the development of T-cell progenitors [66]. The key γδ T-cell-specific stimulus in this protocol is the phosphoantigen HMBPP, which is introduced after the T-cell differentiation stage to stimulate γδ T cells on day 24 of culture in combination with 2-deoxy-D-glucose (2DG) and IL-15. The protocol enables a relatively high yield of the target cell population: starting from 1 × 10^3^ iPSCs, the authors obtained 8 × 10^5^ iγδ T cells. Approximately 70% of the cells had surface CD3. However, intracellular staining demonstrated cytoplasmic CD3 presence in 89% of the cells. The cells did not produce the NK-cell marker NKp46 but did produce CD7. These findings support the conclusion that the resulting population had been differentiated toward the T-cell lineage. All generated cells exhibited comparably high cytotoxic activity. However, functional phenotyping showed that the majority had acquired a terminal effector memory (TEMRA) phenotype. This raises the question of whether these cells would be capable of long-term persistence and proliferation in vivo. The predominance of the TEMRA phenotype indicates a terminally differentiated effector state that may support strong immediate cytotoxic activity but potentially limit the proliferative reserve and long-term persistence of the cells in vivo [110].

Thus, current studies demonstrate the feasibility of generating functional γδ T cells from iPSCs using a variety of differentiation strategies. The early stages of hematopoietic and T-lineage progenitor formation can largely be reproduced using approaches developed for αβ T-cell generation, whereas efficient γδ T-cell development requires additional control of TCR-dependent lineage specification and maturation. Using γδ T cells as the starting material for iPSC generation additionally makes it possible to preserve pre-existing γδ TCR rearrangements and thereby generate cells with predefined TCR specificity. Current technological development is focused on eliminating the need for feeder cells and serum, increasing the yield of the target population, and standardizing culture conditions and the phenotype of the resulting cells. Further development of the technology will require addressing several challenges, including the heterogeneity of the resulting populations, the recapitulation of the physiological spectrum of γδ T-cell phenotypes, preservation of proliferative potential, and long-term persistence of the cells following administration.

#### 3.3.2. Preclinical and Clinical Studies of iγδ T Cells

Various studies have shown that Vγ9Vδ2 T cells exhibit high antitumor potential. This has been demonstrated in both in vitro and in vivo studies on follicular lymphoma [111], skin carcinoma [112], renal cancer [113], prostate cancer [114] and other tumors. In 2008, the possibility of ex vivo expansion of γδ T cells obtained from patients with malignant neoplasms using zoledronic acid and IL-2 was demonstrated for the first time. It was also demonstrated that such cells retain cytotoxic activity. These data made it possible to consider γδ T cells as a new potential population for adoptive immunotherapy [115]. Clinical trials have demonstrated the feasibility of adoptive transfer of ex vivo expanded Vγ9Vδ2T cells to cancer patients, as well as the safety of these cells. In patients with metastatic solid tumors, the feasibility and safety of using autologous ex vivo expanded γδ T cells have also been demonstrated. However, in early clinical studies, the therapeutic effect remained limited [116,117,118,119]. By 2026, more than 90 clinical trials employing γδ T cells of various origins had been registered, and their number continues to grow. The majority of studies focus on the use of γδ T cells for the treatment of a broad spectrum of solid tumors; however, studies on various hematological malignancies also exist (acute myeloid leukemia, acute lymphoblastic leukemia, chronic myeloid leukemia, Hodgkin lymphomas, B-cell non-Hodgkin lymphomas, and others). Two principal approaches are employed: the use of agents to enhance γδ T cell expansion in vivo (by means of γδ T cell agonists, e.g., zoledronate) and the adoptive transfer of ex vivo expanded γδ T cells (predominantly autologous) [120,121]. Historically, the majority of studies have focused on Vγ9Vδ2 T cells; however, interest in Vδ1 T cells has recently been growing (NCT04735471–allogeneic CD20-specific CAR-Vδ1T cells) [122,123]. The limited efficacy of unmodified γδT cells has driven a gradual transition toward the use of CAR-modified γδT cells. This approach allows the non-specific recognition of the γδTCR to be combined with specific recognition by the chimeric receptor (Table 2).

Despite the progress achieved in generating iPSC-derived γδT cells, the published data available to date are limited to preclinical studies. Clinical trials of iγδT cell products have not yet been reported. In 2026, the cytotoxic activity of such cells was demonstrated for the first time in a model of colorectal cancer tumor organoids derived from patient tumor material [110]. The antitumor activity of iγδT cells modified with a bispecific CAR targeting CD19 and CD22 was demonstrated in vitro using the acute lymphoblastic leukemia NALM-6 and diffuse large B-cell lymphoma OCI-Ly1 cell lines, as well as in vivo in the OCI-Ly1 model [124].

Thus, the clinical development of CAR-γδT cells remains predominantly at early stages. The majority of current approaches to generating CAR-modified γδT cells are based on the use of allogeneic cells, reflecting the pursuit of standardized off-the-shelf products. Both hematological malignancy antigens (CD19, CD20) and solid tumor antigens (B7-H3, CD70, PSCA, etc.) are being considered as targets. However, iPSCs have not yet been represented among the cell sources in clinical trials of γδT cell therapy, despite the successful generation of iPSC-derived γδT cells with pronounced antitumor activity in preclinical models. This underscores the potential value of iPSCs as a platform for developing standardized and scalable CAR-γδT products.

### 3.4. iNKT Cells in Cancer Immunotherapy

Natural killer T cells (NKT) are cells that, by their molecular characteristics, reside at the interface of innate and adaptive immunity. These cells carry an antigen-specific T-cell receptor on their surface, which aligns them with classical T cells. However, unlike conventional T cells that recognize antigenic peptides, NKT cells recognize lipids or glycolipids presented in the context of the CD1d molecule, which can be present on several cell types: antigen-presenting cells, double-positive thymocytes, and marginal zone B cells [125,126,127]. The surface of NKT cells also bears markers characteristic of effector T cells and memory T cells. As NKT cells mature, they acquire a number of receptors characteristic of NK cells, including CD161, NKG2D, and certain receptors of the KIR family, although their composition depends on the species, tissue, and stage of differentiation. NKT cells acquire NK cell markers predominantly at late stages of differentiation. iNKT cells, or invariant NKT cells, or type I NKT cells, carry a semi-invariant TCR and are capable of recognizing both self and foreign lipids. Among NKT cells, the invariant cell population is predominant. Their TCR comprises the Vα24-Jα18 and Vβ11 chains in humans and Vα14-Jα18 and Vβ8.2, -7, or -2 in mice. In humans, iNKT cells are represented by three main phenotypes: CD4+, CD8+, and CD4−CD8−. A well-known activating ligand for iNKT cells is α-Galactosylceramide (αGalCer), which activates their proliferation [127,128,129,130]. Like other T cells, iNKT cells develop in the thymus, and they are characterized by passage through a particular variant of selection during differentiation–agonist selection. iNKT precursors, upon passing through the DP stage, recognize CD1d-lipid complexes on other DP thymocytes, which leads to activation of the iNKT-specific differentiation program. In iNKT cell development, there are two checkpoints: the first is associated with passage through selection in the thymus, and the second is associated with the transition of cells to a mature phenotype from NK1.1− (CD161− in humans) to the NK1.1+ stage, which can occur both in the thymus and in the periphery [127,131].

iNKT cells possess a number of advantages in antitumor therapy, and their antitumor efficacy has been confirmed both experimentally and in clinical studies. These advantages are explained by the biology of iNKT cells and by their combination of both innate and adaptive immune functions. The presence of a semi-invariant TCR that recognizes lipid antigens in the context of CD1d—a non-classical molecule similar to MHC class I but, unlike it, monomorphic—enables these cells to simultaneously exhibit the specificity of adaptive immunity and to become rapidly activated, which is characteristic of innate immune cells. Antigen recognition via the CD1d molecule, moreover, offers low propensity to induce graft-versus-host disease upon allogeneic application of the cells. Studies have shown that iNKT cells are capable of recognizing both tumor cells bearing CD1d (specifically via the TCR) and CD1d− tumor cells. It has been demonstrated that, in this case, recognition is mediated in a CD1d-independent yet still TCR-dependent manner. CD1d-independent cytotoxicity is further enhanced by activating NK cell receptors (NKG2D, 2B4, DNAM-1, LFA-1, and CD2), although it does not depend on them entirely. This indicates that iNKT cells are characterized by the simultaneous absence of a number of limitations typical of T cells and NK cells [132]. Furthermore, the uniqueness and value of iNKT cells lie in the demonstrated capacity of these cells to reprogram the tumor microenvironment, to influence myeloid-derived suppressor cells and other immunosuppressive cells, and to promote the restoration of a proinflammatory cell pool within the tumor. Activated iNKT cells produce significant amounts of IFNγ, which activates NK cells. They also promote the activation and proliferation of antigen-specific CD8 and CD4 T cells. In addition, activated iNKT cells promote dendritic cell maturation [133,134,135,136]. Thus, the concerted activation of both innate and adaptive immune mechanisms directed against the tumor occurs and is amplified. It is precisely this feature that makes iNKT cells a promising tool for combating solid tumors. The introduction into the iNKT cell genome of a gene encoding a CAR allows the functionality of these cells to be further expanded and their specificity to be enhanced.

The use of primary iNKT cells is associated with a serious limitation: they constitute at most up to 1% of peripheral blood T cells, may not be detected in it at all, and on average their number amounts to only about 0.1%. Moreover, considerable variability in their number exists among different individuals. Their proportion of adipose tissue T cells reaches 8–12%, while in other tissues, including the liver and spleen, other NKT subpopulations dominate over invariant NKT cells. In patients with oncological diseases, as well as with obesity, their number may decrease substantially. Thus, obtaining iNKT cells in substantial quantities from humans is rather difficult [137,138,139,140]. In this regard, the use of iNKT cells obtained by differentiation from iPSCs is of current relevance.

#### 3.4.1. Differentiation of iPSCs into iNKT Cells

Up to the DP cell stage, the iPSC differentiation into iNKT and αβT cells proceeds along a similar pathway. The key differentiation signal, according to the protocols, is likewise Notch, provided through OP9-DLL1/DLL4 feeder cells, MS5, or through modern feeder-free systems involving the use of plates coated with Notch ligands or microbeads. The natural development of iNKT cells diverges from that of conventional αβT cells at the stage of CD4+CD8+ DP precursor cells. Whereas αβT cells at this stage must undergo positive selection and differentiate toward CD4+ or CD8+ cells, iNKT cells at this stage must undergo rearrangement of the genetic segments encoding the α-chain of the invariant TCR (TRAV10–TRAJ18). These rearrangements and the onset of synthesis of the semi-invariant TCR of iNKT cells confer upon them the ability to recognize glycolipid-CD1d complexes on the surface of neighboring DP thymocytes and the capacity to pass through the agonist selection stage. Subsequently, upon induction of the transcription factor PLZF, the cells develop toward the iNKT lineage [141].

The most convenient and conceptually more simple approach to obtaining iNKT cells is the one employing iPSCs derived from mature primary iNKT cells. In this case, the starting cell already carries a rearranged TCR, and therefore, during differentiation, it is possible to obtain cells with the same iNKT TCR. Moreover, the differentiation potential of such iPSCs into i-iNKT cells is higher due to the phenomenon of epigenetic memory. It is precisely this approach that was used in early studies, and it remains relevant for obtaining an i-iNKT cell product.

The generation of iNKT cells from iPSCs was first demonstrated in 2010, when Hiroshi Watarai et al. obtained NKT cells using mouse cells. In their study, they demonstrated the generation of iPSCs from mouse embryonic fibroblasts carrying functional NKT-specific TCR loci in the germline, as well as from primary NKT cells isolated from the spleen of adult wild-type mice. Subsequently, iPSCs derived from these cells were successfully differentiated into functional iNKT cells that reproduced the known functions of native NKT cells and were capable of suppressing tumor growth in vivo. Successful differentiation was carried out according to a feeder protocol employed for obtaining αβT cells. Based on flow cytometry data, the authors established that the resulting cells phenotypically corresponded rather to early thymic NKT cells, or stage 0 NKT cells: they were represented by double-positive CD4+CD8+ cells bearing T-lymphocyte markers, a TCR corresponding to NKT cells and recognizing α-GalCer in the context of CD1d, and expressed at significant levels the *Zbtb16* gene, which encodes the transcription factor PLZF, a key factor for NKT cell development [142].

The first studies on the generation of human iPSC-derived iNKT (i-iNKT) cells were based on a classical feeder cell-dependent approach. In these studies, iPSCs were generated from primary human Vα24+ iNKT cells. To obtain sufficient numbers of Vα24+ iNKT cells, peripheral blood or cord blood mononuclear cells were cultured in the presence of IL-2 and α-GalCer for one week, followed by magnetic selection of iNKT cells. The isolated cells were then further expanded for four weeks on murine dendritic cells loaded with α-GalCer in the presence of IL-2, IL-7, and IL-15. This approach enabled the generation of sufficient numbers of primary iNKT cells for subsequent reprogramming and iPSC generation [143]. Human i-iNKT cells were subsequently generated in a 2D culture system by differentiating iPSC-derived hematopoietic progenitor cells on OP9/DLL1 cells, followed by supplementation of the culture medium with SCF, IL-7, and FLT3L [143,144]. This approach predominantly yields DN iNKT cells. However, CD4+ iNKT cells are considered particularly promising for ACT because activated CD4+ iNKT cells can contribute to remodeling of the immunosuppressive tumor microenvironment and promote the activation of NK cells, CTLs, and conventional CD4+ T helper cells. In addition, 2D differentiation protocols tend to generate immature iNKT cells that lack the presence of surface protein CD5, a marker associated with mature iNKT cells. To generate more mature iNKT cells with phenotypic and functional characteristics more closely resembling those of primary iNKT cells, differentiation of iPSCs using artificial thymic organoids has been proposed. As in approaches for αβ T-cell differentiation, this technology requires co-culture of iPSCs with murine stromal cells producing the human Notch ligand DLL4. Hematopoietic progenitor cells are first generated from iPSCs, after which a 3D artificial thymic organoid culture is established using MS5 cells producing human DLL4 (MS5/hDLL4) [145].

A current research focus in the field of iPSC-derived iNKT cells is the development of feeder-free and serum-free differentiation protocols. In 2025, Akhilesh Kumar and colleagues proposed such a protocol and demonstrated the generation of i-iNKT cells with phenotypic and functional characteristics resembling those of primary iNKT cells [146].

Importantly, several studies have demonstrated a critical role for specific cytokines, particularly IL-15, in the differentiation of iNKT cells. IL-15 has been shown to be an important factor supporting their maturation and functional development. In its absence, cells may remain at less differentiated stages during development, particularly at the CD4-CD8- DN stage, and may exhibit incomplete expression of key markers associated with the iNKT lineage, including the TCR. When differentiated on OP9/DLL1 feeder cells in the absence of IL-15, the resulting i-iNKT cells exhibited minimal proliferative capacity following specific stimulation with α-GalCer-loaded PBMCs [147,148].

Thus, when primary iNKT cells are used as the starting population for iPSC generation, the subsequent differentiation of these iPSCs into i-iNKT cells follows principles broadly similar to those used for generating iPSC-derived αβ T cells. However, although the presence of IL-15 during the final stages of differentiation is critical for i-iNKT cell maturation, these cells do not require the additional complex maturation procedures used to generate CD4+ or CD8+ conventional iPSC-derived αβ T cells. A similar consideration applies to the expansion of i-iNKT cells. In several protocols, expansion of the differentiated cells can be achieved through the use of cytokines, including IL-2, IL-7, and IL-15. This may potentially simplify the downstream manufacturing process for i-iNKT cell products by reducing the number of technological steps required for their production.

#### 3.4.2. Preclinical and Clinical Studies of i-iNKT Cells

Studies on mouse models provided some of the first evidence for the role of iNKT cells in antitumor immunity. NKT cell-deficient mice (TCR Jα281–deficient) were found to be more susceptible to spontaneous tumor development in models of chemically induced carcinogenesis, and iNKT cells were also shown to participate in the control of metastasis [149]. Preclinical studies have demonstrated antitumor activity of both unmodified iNKT cells and CAR-engineered iNKT cells, including cells expressing genes for anti-GD2 and anti-CD19 CARs [150,151,152]. Importantly, preclinical models have also demonstrated that iNKT cells can limit the development of GvHD [153].

Studies involving patients with malignant diseases have demonstrated an association between reduced numbers of circulating iNKT cells and poorer overall survival. More broadly, across different types of malignancies, including both solid tumors and hematological cancers, it was shown that reduced frequency of iNKT is followed by impaired iNKT-cell function [154,155]. Conversely, higher numbers of these cells in the peripheral blood have been associated with more favorable clinical outcomes [156,157,158]. Collectively, these findings provide further support for the involvement of iNKT cells in antitumor immunity and in the regulation of tumor development and progression.

The possibility of the safe allogeneic use of unmodified iNKT cells was demonstrated in a phase I clinical trial in which these cells were administered to patients with relapsed/refractory multiple myeloma (NCT04754100). The cells were also used in the treatment of patients with severe cases of gastroesophageal adenocarcinoma in a phase II clinical trial (NCT06251793). The product used by the researchers consisted of ex vivo expanded allogeneic iNKT cells obtained from healthy donors and administered to patients. Studies demonstrated a favorable safety profile of these cells: no clinically significant GvHD was observed, and neither severe CRS nor ICANS occurred [159].

The efficacy and safety of CAR-NKT as well as CAR-iNKT cells are also being evaluated in clinical trials. For many studies, the specific cell population on which the product is based (type I or type II NKT cells) is not specified. As of September 2026, a total of nine clinical trials of these cells have been registered (Table 3). Both autologous and allogeneic approaches to the manufacturing of these cells are being investigated. Some studies are focused on the use of these cells against hematological CD19-positive malignancies (NCT05487651, NCT03774654, NCT04814004, NCT07518329), whereas others are focused on their use against solid GD2-positive and CD70-positive tumors (NCT03294954, NCT06182735, NCT06394622, NCT06728189, NCT06870279).

Studies of iPSC-derived iNKT cells have progressed from the preclinical stage to the first clinical trials involving patients (Table 3). The results of the first clinical study of iPSC-derived iNKT cells were published in 2025. The study included 10 patients with relapsed head and neck cancer; the clinical product consisted of allogeneic iPSC-derived iNKT cells, which were administered into the artery supplying the tumor. Adverse events were observed in one of the 10 patients, while tumor progression was suppressed in two patients. These patients also showed an increase in the number of CD8+ T cells with memory and effector phenotypes. These findings provided the first evidence of the safety and potential antitumor activity of i-iNKT cells (jRCT2033200116) [160]. In 2026, the study protocol for a new clinical trial of i-iNKT cells was published (jRCTa030220741). The authors plan to administer i-iNKT cells intra-arterially in combination with the administration of DC/Gal (dendritic cells treated with α-galactosylceramide) into the nasal submucosal layer. The study is designed to evaluate tolerability and safety, as well as immunological changes occurring following treatment [161].

Thus, the body of preclinical and clinical evidence indicates that iNKT cells have substantial potential for antitumor therapy. Their ability to directly eliminate tumor cells, activate other components of both innate and adaptive immunity, and modulate the tumor microenvironment provides a multifaceted mechanism of antitumor activity. At the same time, the low frequency of iNKT cells in peripheral blood substantially limits the ability to obtain large numbers of autologous cells, while their functional characteristics may vary among patients with malignant diseases. Preclinical and early clinical studies indicate a potentially favorable safety profile of iNKT-cell products, including a low propensity for the development of GvHD and severe toxicities characteristic of some other types of T-cell therapies. The generation of iNKT cells from iPSCs represents the next stage in the development of this field, as it enables the establishment of a virtually unlimited source of cells with defined characteristics and potentially allows the production of a standardized allogeneic product. The emergence of the first clinical data on iPSC-derived iNKT cells indicates that this technology has entered the stage of clinical translation; however, assessment of its antitumor efficacy is currently based on a limited number of patients and requires confirmation in subsequent studies.

### 3.5. MAIT Cells in Cancer Immunotherapy

Mucosal-associated invariant T (MAIT) cells are a subset of innate-like αβ T cells characterized by a restricted TCR repertoire. Relatively large numbers of these cells are present in the lamina propria of the lungs, liver, and intestine. Approximately 1–10% of MAIT cells circulate in the peripheral blood. In humans, these cells express an invariant TCR Vα7.2-Jα33 chain paired with a restricted repertoire of TCR β chains, frequently Vβ2/Vβ13. A key feature distinguishing these cells from conventional αβ T cells is their non-canonical mode of antigen recognition. Antigen recognition is restricted by the MR1 (major histocompatibility complex class I-related) molecule, which predominantly presents riboflavin biosynthesis metabolites, such as 5-OP-RU, 5-OE-RU, and 1H,3H,5-A-RU [162].

Upon activation, these cells have been shown to exhibit high cytotoxic activity mediated by perforin and granzymes, as well as the ability to secrete various cytokines, including IFN-γ, TNF-α, GM-CSF, and IL-17. In addition, they have activating NK-cell receptors and TLRs on their surface [163,164]. The tropism of these cells for peripheral tissues and their ability to actively migrate into these tissues, in addition to the other features described above, make them promising cells for the immunotherapy of solid tumors. Introduction of a gene encoding a CAR can confer defined antigen specificity on these cells, thereby potentially enhancing their antitumor properties.

In peripheral blood, MAIT cells are relatively abundant and account for approximately 1–10% of circulating T cells, with substantial interindividual variability. As for these cells, the main limitation for ACT using ex vivo-expanded primary MAIT cells is their relatively low proliferative capacity. This limitation can be overcome using artificial antigen-presenting cells loaded with MAIT-specific antigens; however, this approach is considerably less convenient than the conventional approach to the expansion of conventional T cells using cytokines [162,165]. Therefore, approaches for generating MAIT cells from iPSCs are being developed, which may enable the production of virtually unlimited numbers of target cells. When an iPSC clone generated from a mature primary MAIT cell is used, the differentiation protocols are fundamentally similar to those applied to other T-cell types. Such protocols enable sufficiently high cell yields to be obtained; however, the generation of cells with a sufficiently mature phenotype remains a challenge. In vivo studies have shown that this limitation is overcome following administration of iMAIT cells to animals. Following in vivo administration, iMAIT cells successfully undergo maturation [166]. Studies in animals have also demonstrated that, following adoptive transfer, murine iPSC-derived MAIT cells actively migrate to various organs, exhibit cytotoxic activity, suppress the formation of lung metastases, and increase animal survival [167].

In vitro studies have demonstrated the possibility of generating CAR-MAIT cells from primary cells. High cytotoxicity has been demonstrated for CD19-CAR-MAIT, HER2-CAR-MAIT, and mesothelin-CAR-MAIT cells. However, most studies to date have been limited to in vitro conditions [168,169].

MAIT cells represent a promising type of effector T lymphocyte for the development of universal cell therapy products because they combine a restricted TCR repertoire, predominantly MR1-dependent antigen recognition, low alloreactivity, and pronounced cytotoxic properties. These features make MAIT cells potentially attractive for the development of allogeneic and off-the-shelf products, including in combination with CAR technology. The generation of MAIT cells from iPSCs may overcome limitations associated with the relatively low availability and variability of primary MAIT cells and potentially provide a virtually unlimited source of standardized cells. MAIT-derived iPSCs have been shown to differentiate into cells with characteristic phenotypic and functional features of MAIT cells; however, the resulting reMAIT cells remain less mature than peripheral MAIT cells in many respects. The most convincing evidence of the antitumor activity of iPSC-derived MAIT cells has been obtained predominantly in preclinical models, whereas the efficacy of human iPSC-MAIT cells specifically in cancer therapy remains insufficiently studied. iPSC-MAIT cells represent a promising but still poorly characterized platform for the development of universal antitumor cell therapy, the potential of which remains to be confirmed in clinical studies.

### 3.6. Safety and Quality Control of iPSC-Derived T-Cell Products

In addition to differentiation efficiency, phenotypic maturation, and manufacturing scalability, the clinical translation of iPSC-derived T-cell products requires rigorous safety and quality assessment. One of the major concerns is the potential presence of residual undifferentiated iPSCs or aberrantly differentiated cells in the final product. Indeed, incomplete iPSC differentiation represents a potential tumorigenic risk. This concern is particularly relevant to manufacturing platforms involving prolonged culture, multiple differentiation stages, and extensive cell expansion. Accordingly, residual pluripotent cells should be considered a critical impurity, and sensitive, product-specific assays should be incorporated into quality control strategies. Approaches such as flow cytometry for pluripotency-associated markers and molecular assays, including quantitative PCR or digital PCR, may be used to detect residual undifferentiated cells. In vivo tumorigenicity studies may also be required depending on the product and its risk profile [170,171].

Another important consideration is genomic integrity throughout the manufacturing process. Genetic abnormalities may be present in parental somatic cells, arise during reprogramming, or become enriched during prolonged iPSC culture and clonal expansion [172]. Genome editing can be the cause of additional potential risks, including off-target modifications, unintended on-target changes, chromosomal rearrangements, and selection of clones carrying undesirable genetic alterations [173]. Such changes may affect cell growth, differentiation potential, functional stability, or tumorigenic risk. Therefore, genomic characterization should be performed at appropriate stages of product development, including the establishment and qualification of the parental iPSC line and, where applicable, the selection of genetically modified clones. Importantly, no single assay can exclude all potentially relevant abnormalities; a risk-based combination of complementary methods is therefore necessary.

The identity, purity, and functional potency of the final T-cell product must also be defined using product-specific critical quality attributes. Identity testing should confirm the intended T-cell lineage and, where applicable, the presence of CAR. Purity assessment should quantify the intended population and identify potentially undesirable cells, including residual pluripotent cells, off-target differentiated populations, and contaminating non-T cells. Product-specific criteria are particularly important when obtaining iPSC-derived αβ T, γδ T, iNKT, and MAIT cells, because their antigen-recognition mechanisms and characteristic phenotypes differ. Thus, lineage-associated markers, as well as antigen-recognition properties and functional properties, must be considered.

Functional potency should be assessed including tests for cytotoxicity, cytokine production, and appropriate measures of activation or persistence. For genetically engineered products, assays should also address the function of the introduced CAR and, where relevant, the intended effects of additional genetic modifications. A combination of validated analytical methods and functional assays is therefore required to demonstrate batch consistency and to support the release of a product for clinical use.

The development of iPSC-derived T-cell therapies requires predefined acceptance criteria for product identity, purity, potency, genomic integrity, sterility, and other relevant safety attributes, with the specific testing strategy determined by the product’s characteristics and manufacturing process. A fairly broad range of tests and methods should be employed to assess the quality and safety of iPSC-derived products intended for clinical use, and scalable iPSC-derived T cell production must be accompanied by a risk-based release strategy [174].

## 4. Conclusions

Thus, the efficacy of autologous CAR T-cell therapy has already been demonstrated and established worldwide. At the same time, the limitations and challenges associated with this therapeutic approach have been identified and described, including variability in the starting cellular material, its functional state, the duration of manufacturing, and the inability to manufacture a therapeutic product for some patients with malignant neoplasms. In the context of adoptive cancer therapy, iPSC technology may overcome a number of limitations associated with the use of autologous primary cells. The virtually unlimited capacity for genetic modification of iPSCs and their derivatives, together with the ability to manufacture and expand T-cell populations that are present in the body at very low frequencies (γδ T, iNKT, and MAIT cells), may enable the precise selection of cellular phenotypes that provide optimal persistence and expansion in the patient, migration to tumor tissue, and antitumor activity mediated through mechanisms of both innate and adaptive immunity. Appropriate application of iPSC technology may also help overcome limitations associated with undesirable adverse events, including cytokine release syndrome and GvHD.

Moreover, different T-cell subsets possess potentially complementary mechanisms of antitumor activity, which may be particularly relevant for the treatment of solid malignancies. Thus, αβ T cells possess pronounced mechanisms of antigen-specific responses, whereas γδ T, iNKT, and MAIT cells combine mechanisms characteristic of adaptive immune responses with those associated with innate immunity. Depending on the subset, γδ T cells combine specific recognition of phosphoantigens, lipid molecules, and other antigens through the TCR with the production of a range of NKRs, TLRs, and the antibody-dependent cellular cytotoxicity receptor CD16. In addition to their semi-invariant TCR and NKRs, iNKT cells have the unique ability to activate other immune cells within the tumor microenvironment, thereby overcoming the immunosuppressive effects exerted by tumor cells and other cell populations within it. MAIT cells exhibit a high capacity for migration to various organs and high cytotoxic activity. Overall, non-conventional T cells, especially iNKT, may be more applicable for the treatment of solid tumors as they are more likely to enter the tumor microenvironment and are able to reprogram it and possess MHC-independent cytotoxicity. Thus, the biology of different T-lymphocyte subpopulations may help address various limitations associated with conventional CAR T-cell therapy based on primary T cells (Table 4). These complementary mechanisms of antitumor activity may pave the way for new combined iPSC-cell-based therapies involving the use of different T-cell subsets as it may help address different complications associated with the treatment of malignant tumors.

At the same time, the data currently available indicate that, although several technologies based on iPSC-derived CAR T cells have reached the clinical trial stage, this field requires further development. This is due to the persistence of several challenges, including the functional maturation of the resulting cells, the phenotype of the generated cells, and the need to optimize feeder-free and serum-free protocols to enable full clinical application and scalable manufacturing. A list of methods necessary for extensive quality and safety control must also be identified for broad clinical use of iPSC-derived CART cell products.

iPSC technology may ultimately serve as a platform for the engineering of CAR T-cell products in which cells with defined antigen specificity, phenotype, effector functions, and immune defense mechanisms can be selected according to specific therapeutic objectives.

## Figures and Tables

**Table 1:** Clinical studies involving iPSC-derived αβ CAR T cells. The table summarizes early-phase clinical studies of allogeneic iPSC-derived αβ T-cell products, including their target antigens, intended therapeutic indications, clinical development phase, and genetic modifications.

Trial ID	Target	Disease	Phase	Genetic Modifications and Their Intended Functions
NCT04629729	CD19	BCL, CLL, Precursor B-ALL	Phase I	CD19 CAR under the regulation of the TRAC locus to delete TCR [79]
NCT06241456	HER2	HER2-positive or other advanced solid tumors	Phase I	HER2 CAR TCR null CD38 null IL7RF TGFβSRR CXCR2 hnCD16a
NCT07216105	MICA/B—monotherapy EGFR-targeting mAb cetuximab or HER-2 targeting mAb trastuzumab—in combination	Advanced solid tumors	Phase I	MICA/B CAR targeting the conserved a3 domain hnCD16a FC for multi-antigen targeting ADR expression CD58 KO CXCR2 and TGFb signal redirection receptors to maximize tumor trafficking and adaptability within tumor niche [80]
NCT07221032	MICA/B–monotherapy CD38-targeting mAb daratumumab	Relapsed and/or Refractory MM	Phase 1	MICA/B CAR targeting the conserved a3 domain hnCD16a FC for multi-antigen targeting ADR expression CD58 KO CXCR2 and TGFb signal redirection receptors to maximize tumor trafficking and adaptability within tumor niche

**Abbreviations:** Abbreviations: ADCC, antibody-dependent cellular cytotoxicity; ALL, acute lymphoblastic leukemia; B-ALL, B-cell acute lymphoblastic leukemia; BCL, B-cell lymphoma; CAR, chimeric antigen receptor; CLL, chronic lymphocytic leukemia; CXCR2, C-X-C motif chemokine receptor 2; HER2, human epidermal growth factor receptor 2; hnCD16a, high-affinity non-cleavable CD16a; IL7RF, interleukin-7/interleukin-7 receptor fusion protein; iPSC, induced pluripotent stem cell; MICA/B, MHC class I polypeptide-related sequence A/B; MM, multiple myeloma; TCR, T-cell receptor; TGFβ-SRR, transforming growth factor-β signal redirection receptor; TRAC, T-cell receptor alpha constant gene.

**Table 2:** Clinical studies involving CAR-engineered γδ T cells. The table summarizes clinical studies of γδ T-cell products, including their target antigens and combination regimens, intended therapeutic indications, and clinical development phase.

Drug	Cell Source	Disease	Phase	Trial ID
CD7 CAR γδ T Fludarabine Cyclophosphamide	Allogeneic	Relapsed/Refractory T-ALL/LBL Relapsed/Refractory AML	Phase I	NCT07120607
CD20 CAR γδ T	Allogeneic	FL MCL MZL PMBCL DLBCL NHL	Phase I	NCT04911478
CD7 CAR γδ T	-	Adult Relapsed/Refractory Hematologic and Lymphatic Systemic Malignancies	Early Phase 1	NCT07338604
CD7 CAR γδ T	-	T Cell-derived Malignant Tumors	Early Phase 1	NCT04702841
HLA-G-CAR, PD-L1/CD3ε BiTE secreting γδ T	Allogeneic	Relapsed/Refractory Solid Tumors	Phase 1 Phase 2	NCT06150885
CD19 CAR γδ T Cyclophosphamide Fludarabine	Allogeneic	Relapsed/Refractory CD19-positive B-ALL	Phase 1	NCT07491263
NKG2DL CAR γδ T	Allogeneic	Colorectal Cancer Triple Negative Breast Cancer Sarcoma Nasopharyngeal Carcinoma Prostate Cancer Gastric Cancer	Phase 1	NCT04107142
B7H3 CAR γδ T	Allogeneic	Solid Tumor Meningeal Metastasis	N/A	NCT06592092
CD70 CAR, dominant negative TGFβ receptor II expressing Vδ1 γδ T cells Fludarabine Cyclophosphamide	Allogeneic	Relapsed/Refractory Clear Cell Renal Cell Carcinoma	Phase 1 Phase 2	NCT06480565
CD20 CAR Fludarabine Cyclophosphamide	Allogeneic	FL MCL MZL PMBCL DLBCL NHL	Phase 1	NCT04735471
NKG2DL CAR γδ T	Allogeneic	Advanced Solid tumors or hematological Malignancies	Phase 1	NCT05302037
PSMA CAR Vδ1 γδ T cells Fludarabine Cyclophosphamide	Allogeneic	Metastatic Castration-Resistant Prostate Cancer	Phase 1 Phase 2	NCT07793435

**Abbreviations:** Abbreviations: T-ALL/LBL, T-cell acute lymphoblastic leukemia/lymphoma; AML, acute myeloid leukemia; FL, follicular lymphoma; MCL, mantle-cell lymphoma; MZL, marginal zone lymphoma; PMBCL, primary mediastinal large B-cell lymphoma; DLBCL, diffuse large B-cell lymphoma; NHL, non-Hodgkin lymphoma; B-ALL, B-cell acute lymphoblastic leukemia; BiTE, bispecific T-cell engager.

**Table 3:** Clinical studies involving CAR-NKT and i-iNKT. The table summarizes early-phase clinical studies of autologous as well as allogeneic CAR-NKT and iPSC-derived iNKT-cell products. Intended clinical applications and target antigens are listed.

Product Type	Cell Type	Drug	Cell Source	Disease	Trial ID
CAR-modified cells	NKT type I (iNKT) cell product	CD19 CAR, IL-15 armored	Allogeneic	Relapsed/Refractory/High-risk B-cell Tumors	NCT04814004
		CD19 CAR	Allogeneic	NHL CLL HCL	NCT07518329
	NKT cell product (cell type not specified)	CD70 CAR	Autologous	Advanced Malignant Solid Tumors	NCT06728189
		CD70 CAR NKT cells Cyclophosphamide + Fludarabine	Autologous	Advanced Malignant Solid Tumors	NCT06394622
		CD70 CAR NKT cells Cyclophosphamide + Fludarabine	Autologous	RCC	NCT06182735
		CD70-targeted CAR-NKT	Autologous	Advanced Clear Cell RCC	NCT06870279
		CD19 CAR	Allogeneic	Refractory NHL Refractory B-Cell Small Lymphocytic Lymphoma Relapsed Adult ALL Relapsed CLL Relapsed NHL	NCT03774654
		CD19 CAR Interleukin-15	Allogeneic	NHL, Relapsed, Adult B-cell Lymphoma B-cell Leukemia DLBCL ALL, Adult B Cell ALL, Childhood CLL/SLL	NCT05487651
		GD2 CAR, IL-15 expressing NKT	Autologous	Neuroblastoma	NCT03294954
iPSC-derived iNKT cells	NKT type I (iNKT) cell product	Arterial infusion of iPSC-iNKT cells	Allogeneic	Recurrent head and neck cancer	jRCT2033200116
		Arterial infusion of iPSC-iNKT cells Nasal submucosal DC/Gal injection	Allogeneic	Recurrent and advanced head and neck cancer	jRCTa030220741

**Abbreviations:** CAR, chimeric antigen receptor; NHL, non-Hodgkin lymphoma; CLL, chronic lymphocytic leukemia; HCL, hairy cell leukemia; RCC, renal cell carcinoma; ALL, acute lymphoblastic leukemia; DLBCL, diffuse large B-cell lymphoma; SLL, small lymphocytic lymphoma.

**Table 4:** Characteristics of different T cell subsets.

Characteristic	αβ-T	γδ-T	iNKT	MAIT
Antigen-recognition mechanisms	MHC class I (CD8+ T cells) or MHC class II (CD4+ T cells) restricted. TCR comprising α- and β-chain TCR recognizes peptide molecules	Non-MHC restricted. TCR comprising γ- and δ- chains. Distinct subsets can recognize peptides, phosphoantigens, lipid and glycolipid molecules. Recognition of stress-induced and “missing-self” ligands	CD1d-dependent recognition of lipid and glycolipid molecules. TCR-Independent activation through cytokines a capacity to recognize CD1D-negative tumor cells has been shown	MR1-dependent. Primarily recognize riboflavin metabolites
Tissue infiltration capacity	Dependent on chemokine receptors and adhesion molecules on cells. Most of primary αβ-T reside in blood and lymphoid tissues	Definite tropism of primary cells to peripheral tissues such as skin, intestine, liver and others	A small number of cells reside in blood, most are found in peripheral tissues, such as adipose tissue	Most cells are present in the lamina propria of the lungs, liver, and intestine, 1–10% circulate in blood
Tumor-targeting capacity	High, provided a suitable TCR or CAR is present. High clinical validation, especially for CD19- and BCMA-positive hematologic malignancies. Restricted for solid tumors	High, provided by TCR as well as NK-cell receptors. CAR-engineering allows increasing tumor-targeting capacity	High, provided by TCR as well as innate recognition receptors. Proven ability to reprogram tumor microenvironment	Potentially high
Alloreactivity and GvHD risk	Significant risk associated with the presence of an endogenous alloreactive TCR. For “off-the-shelf” products, strategies to eliminate or suppress the TCR and manage immunogenicity are required	Relatively low risk due to MHC-independent recognition	Relatively low risk due to MHC-independent recognition	Relatively low risk due to MHC-independent recognition
iPSC-to-T cell differentiation efficiency, manufacturing scalability	High. Requires further improvement for CD4+ T-cell differentiation. Protocols were developed that ensure relatively high scalability	High, yet the area is less mature. Needs improvement in the context of heterogeneity of the resulting populations, preservation of proliferative potential, and long-term persistence of resulting cells	High. Approaches for the scalable production of i-iNKT cells have been developed	The feasibility of obtaining MAIT cells and iPSCs has been demonstrated, yet the technology is currently the least mature
Clinical evidence on iPSC-derived cells	There are clinical trials of allogeneic iPSC-derived CAR-T cells, although most of these studies are at the early stages	Currently no clinical data on iPSC-derived γδ-T and CAR γδ-T cells	Technology for i-iNKT cells has entered early stages of clinical translation, however, without CAR-engineering. Antitumor efficacy requires confirmation in subsequent studies	There are no clinical data; development is currently at the preclinical stage

## Data Availability

No new data were created or analyzed in this study. Data sharing is not applicable to this article.

## Abbreviations

The following abbreviations are used in this manuscript:

ACT	Adoptive cell therapy
ADCC	Antibody-dependent cellular cytotoxicity
ALL	Acute lymphoblastic leukemia
AML	Acute myeloid leukemia
ATO	Artificial thymic organoid
BCMA	B-cell maturation antigen
B-ALL	B-cell acute lymphoblastic leukemia
CAR	Chimeric antigen receptor
CLL	Chronic lymphocytic leukemia
CLP	Common lymphoid progenitor
CRS	Cytokine release syndrome
CTL	Cytotoxic T lymphocyte
DGK	Diacylglycerol kinase
DLL1	Delta-like ligand 1
DLL4	Delta-like ligand 4
DN	Double-negative
DP	Double-positive
DLBCL	Diffuse large B-cell lymphoma
FL	Follicular lymphoma
FLT3L	FMS-like tyrosine kinase 3 ligand
GvHD	Graft-versus-host disease
HCL	Hairy cell leukemia
HLA	Human leukocyte antigen
HMBPP	(E)-4-Hydroxy-3-methyl-but-2-enyl pyrophosphate
HSC	Hematopoietic stem cell
ICANS	Immune effector cell-associated neurotoxicity syndrome
iCAR T	Induced pluripotent stem cell-derived chimeric antigen receptor T cell
iγδ T	Induced pluripotent stem cell-derived γδ T cell
iNKT	Invariant natural killer T cell
iPSC	Induced pluripotent stem cell
iT	Induced pluripotent stem cell-derived T cell
IPP	isopentenyl pyrophosphate
MCL	Mantle cell lymphoma
MZL	Marginal zone lymphoma
MAIT	Mucosal-associated invariant T cells
MHC	Major histocompatibility complex
MIC	MHC class I chain-related molecules
MM	Multiple myeloma
NKR	Natural killer cell receptor
NK	Natural killer
NKT	Natural killer T cell
NHL	Non-Hodgkin lymphoma
PBMCs	Peripheral blood mononuclear cells
PMBCL	Primary mediastinal large B-cell lymphoma
PMA	Phorbol 12-myristate 13-acetate
RCC	Renal cell carcinoma
SCF	Stem cell factor
SDF-1	Stromal cell-derived factor 1
SP	Single-positive
TCR	T-cell receptor
TEC	Thymic epithelial cells
T_CM_	Central memory T cell
T_EM_	Effector memory T cell
T_EMRA_	Terminally differentiated effector memory T cell
T_EFF_	Effector T cell
TLR	Toll-like receptor
T_N_	Naïve T cell
TPO	Thrombopoietin
T_REG_	Regulatory T cell
T_SCM_	Stem cell memory T cell
VCAM1	Vascular cell adhesion molecule 1
